# Reply to Kratz et al.

**DOI:** 10.1038/s41431-020-00710-y

**Published:** 2020-08-13

**Authors:** Thierry Frebourg, Svetlana Bajalica Lagercrantz, Carla Oliveira, Rita Magenheim, D. Gareth Evans, the European Reference Network GENTURIS, Nicoline Hoogerbrugge, Marjolijn Ligtenberg, Rianne Oostenbrink, Rolf Sijmons, Emma Woodward, Marc Tischkowitz, Eamonn Maher, Rosalie E. Ferner, Stefan Aretz, Isabel Spier, Verena Steinke-Lange, Elke Holinski-Feder, Evelin Schröck, Thierry Frebourg, Claude Houdayer, Chrystelle Colas, Pierre Wolkenstein, Vincent Bours, Eric Legius, Bruce Poppe, Kathleen Claes, Robin de Putter, Ignacio Blanco Guillermo, Gabriel Capella, Joan Brunet Vidal, Conxi Lázaro, Judith Balmaña, Hector Salvador Hernandez, Manuel Teixeira, Emma Tham, Lubinski Jan, Karolina Ertmanska, Bela Melegh, Mateja Krajc, Ana Blatnik, Sirkku Peltonen, Marja Hietala

**Affiliations:** 1 Department of Genetics, Rouen University Hospital and Inserm U1245, Normandy Centre for Genomic and Personalized Medicine, Normandie University, UNIROUEN, Rouen, France; 2 Hereditary Cancer Unit, Department of Clinical Genetics, Karolinska University Hospital, Stockholm, Sweden; 3 i3S—Instituto de Investigação e Inovação em Saúde & Institute of Molecular Pathology and Immunology of the University of Porto, and Porto Comprehensive Cancer Center, Porto, Portugal; 4 Community Representative, Berlin, Germany; 5 Division of Evolution and Genomic Sciences, Manchester Centre for Genomic Medicine, University of Manchester, MAHSC, St Mary’s Hospital, Manchester University Hospitals NHS Foundation Trust, Manchester, UK; 6 Genomic Medicine, Central Manchester Foundation Trust, Manchester, UK; 7 Department of Human Genetics, Radboud University Medical Center, Nijmegen, The Netherlands; 8 Erasmus Medical Center, Rotterdam, The Netherlands; 9 University Medical Center, Groningen, The Netherlands; 10 Genomic Medicine, Central Manchester Foundation Trust, Manchester, UK; 11 Department of Medical Genetics, National Institute for Health Research Cambridge Biomedical Research Centre, Cambridge University Hospital NHS Foundation Trust, Cambridge, UK; 12 Guy’s and St. Thomas’ NHS Foundation Trust, London, UK; 13 University Hospital Bonn, Bonn, Germany; 14 Medizinisch Genetisches Zentrum, Munich, Germany; 15 Hereditary Cancer Syndrome Center Dresden, Dresden, Germany; 16 Department of Genetics, Rouen University Hospital and Inserm U1245, Normandie University, UNIROUEN, Normandy Centre for Genomic and Personalized Medicine, Rouen, France; 17 Department of Genetics, Institut Curie, Paris Sciences et Lettres Research University, Paris, France; 18 University Hospital Henri Mondor-National Referral Center, Créteil, France; 19 University Hospital, Liege, Belgium; 20 University Hospital Leuven, Leuven, Belgium; 21 Ghent University Hospital, Ghent, Belgium; 22 lnstitut Catala d’Oncologia, Hospital Universitari Germans Trias i Pujol y ICO Badalona, Barcelona, Spain; 23 Hospital Sant Joan de Déu, Barcelona, Spain; 24 i3S- Instituto de Investigação e Inovação em Saúde & Institute of Molecular Pathology and Immunology of the University of Porto, and Porto Comprehensive Cancer Center, Porto, Portugal; 25 Hereditary Cancer Unit, Department of Clinical Genetics, Karolinska University Hospital, Stockholm, Sweden

**Keywords:** Risk factors, Genetics

## To the Editor:

We thank Kratz et al. for their constructive comments which are mostly focused on the differences with the guidelines elaborated in the framework of an international consortium coordinated by Canadian and US teams in 2017 [1].

In medical genetics, the paradigm of cystic fibrosis and *CFTR*-related disorders has shown that it may be appropriate, not only for health professionals but also for patients, to expand the definition of a syndrome to a wider molecularly based definition, in order to highlight the diversity of phenotypes associated with germline variants. Therefore, we think that it is indeed appropriate to expand the Li–Fraumeni syndrome (LFS) toward to a wider and molecularly based cancer predisposition syndrome, designated heritable *TP53-*related cancer syndrome. We agree that the recommendation of testing patients presenting only jaw osteosarcoma is so far not supported by published articles but only, as we indicated [2], on the experience of certain centres. Whereas we considered that it was not justified at the present time to systematically test all children with osteosarcoma (the mutation detection rate being estimated up to 3.8% [3]), the recurrent identification of germline disease-causing *TP53* variants in patients with jaw osteosarcoma, an infrequent location as compared to long bones, lead us to formulate this recommendation. It seems that our colleagues have overinterpreted the statement “Testing for disease-causing *TP53* variants should be performed before starting treatment in order to avoid in variant carriers, *if possible*, radiotherapy and genotoxic chemotherapy and to prioritize surgical treatments.” We fully agree that, in cancer patients carrying disease-causing *TP53* variants, the first priority is to effectively treat the tumours but we believe that a multidisciplinary team should discuss the risks of recurrence and subsequent primary tumours before the initiation of treatment and choose the best therapy. For instance, after identification of a germline *TP53* disease-causing variant in a young woman with invasive, T1N0 breast cancer mastectomy should be offered instead of breast-conserving surgery followed by radiotherapy. The previously published guidelines [1] recommend performing (in all germline *TP53* variant carriers), a medical follow-up including annual whole-body MRI (WBMRI) and brain MRI starting from the first year of age, independently of the personal and medical history and type of *TP53* variant. However, we must now recognize that the global penetrance of germline *TP53* variants has been overestimated, likely depending on so far unrecognized modifying factors. More importantly, we must be aware that only a minor fraction of germline *TP53* variant carriers worldwide, and in particular in the USA, have currently access to this intensive protocol. In our guideline, we advocate for a stratified strategy, by recommending presymptomatic testing and the intensive protocol in childhood from birth, under the following conditions: “the index case has developed a childhood cancer; or childhood cancers have been observed within the family; or this variant has already been detected in other families with childhood cancers; or this variant corresponds to a dominant-negative missense variant.” However, we also carefully open the door by indicating that testing children in families with only early-onset adult cancers can be considered, but only after careful discussion with the parents in order to address the burden, and uncertain benefits, of surveillance in childhood [2]. Our colleagues consider that the surveillance interval that we propose in children for the detection of adrenocortical carcinoma is too long, compared to the interval previously recommended (6 vs. 3–4 months). They may be right (especially until the age 5, probably not above the age 10), but we are not aware of studies demonstrating the additional value of performing a follow-up every 3–4 months. All the studies, except one, published so far and reporting the efficiency in *TP53 v*ariant carriers of WBMRI, in terms of tumour detection rate, have been performed without Gadolinium enhancement, which leads to this recommendation [2]. In females with germline disease-causing *TP53* variants, breast cancer risk increases significantly after the second decade with a peak between 30–44 years and cumulative risk reaches a plateau before 60 [4–6]. Therefore, we think that it is appropriate to fix an age limit for breast MRI at 65 years. A recent review on brain tumours in *TP53* variant carriers has confirmed that brain tumours present a bimodal distribution with the highest peak in young children before 5 years of age and a small peak in adults observed between the third and fourth decades. This supports our proposal to perform brain MRI until 50 years [7]. Finally, we confirm that the studies, which had suggested that colorectal cancer (CRC) is associated with germline *TP53* variants, suffer from certain limitations: the first [8] reported in a series of 397 patients, from 64 LFS families, 16 cases of CRC (4%). The lifetime risk for CRC is estimated in the general population to 4%. Furthermore, among the patients with CRC, the majority had not been tested themselves but were first- or second-degree relatives of *TP53* variant carriers. In a second article [9], the authors reported in a series of 467 patients with CRC at age 40 years or younger, six germline *TP53* variants but examination of these variants, based on the current classification criteria, shows that only two out of the six variants meet criteria for being classified as a class 4 or 5 variant. A third study [10] reported colorectal tumours in 8 among 93 patients with germline *TP53* variants (8.6%), but the authors did not provide data on *TP53* variants. As cancer geneticists and oncologists, we highlight that the risk of overloading the medical follow-up in high genetic risk individuals is to alter the compliance of the patients.

In conclusion, we do not think that the European guidelines elaborated by the ERN GENTURIS, that have been developed with an active participation of patient representatives [2], are in opposition to the guidelines previously published [1]. They instead constitute a stratified version of the previous ones, which may be easier to implement in different countries for patient benefits.

## References

[1] Kratz CP, Achatz MI, Brugières L, Frebourg T, Garber JE, Greer MC (2018). Cancer screening recommendations for individuals with Li-Fraumeni syndrome. Clin Cancer Res.

[2] Frebourg T, Bajalica Lagercrantz S, Oliveira C, Magenheim R, Evans DG. European Reference Network GENTURIS. Guidelines for the Li-Fraumeni and heritable *TP53*-related cancer syndromes. Eur J Hum Genet. 2020. 10.1038/s41431-020-0638-4. 10.1038/s41431-020-0638-4 PMC7609280 32457520

[3] Mirabello L, Yeager M, Mai PL, Gastier-Foster JM, Gorlick R, Khanna C (2015). Germline TP53 variants and susceptibility to osteosarcoma. J Natl Cancer Inst.

[4] Mai PL, Best AF, Peters JA, DeCastro RM, Khincha PP, Loud JT (2016). Risks of first and subsequent cancers among TP53 mutation carriers in the National Cancer Institute Li-Fraumeni syndrome cohort. Cancer.

[5] Amadou A, Waddington Achatz MI, Hainaut P (2018). Revisiting tumor patterns and penetrance in germline TP53 mutation carriers: temporal phases of Li-Fraumeni syndrome. Curr Opin Oncol.

[6] Shin SJ, Dodd-Eaton EB, Peng G, Bojadzieva J, Chen J, Amos CI (2020). Penetrance of different cancer types in families with Li-Fraumeni syndrome: a validation study using multi-center cohorts. Cancer Res..

[7] Orr BA, Clay MR, Pinto EM, Kesserwan C (2020). An update on the central nervous system manifestations of Li-Fraumeni syndrome. Acta Neuropathol..

[8] Wong P, Verselis SJ, Garber JE, Schneider K, DiGianni L, Stockwell DH (2006). Prevalence of early onset colorectal cancer in 397 patients with classic Li-Fraumeni syndrome. Gastroenterology.

[9] Yurgelun MB, Masciari S, Joshi VA, Mercado RC, Lindor NM (2015). Germline TP53 mutations in patients with early-onset colorectal cancer in the colon cancer family registry. JAMA Oncol.

[10] MacFarland SP, Zelley K, Long JM, McKenna D, Mamula P, Domchek SM (2019). Earlier colorectal cancer screening may be necessary in patients with Li-Fraumeni syndrome. Gastroenterology.

